# An agent-based model of the population dynamics of *Anopheles gambiae*

**DOI:** 10.1186/1475-2875-13-424

**Published:** 2014-11-05

**Authors:** SM Niaz Arifin, Ying Zhou, Gregory J Davis, James E Gentile, Gregory R Madey, Frank H Collins

**Affiliations:** Department of Computer Science and Engineering, University of Notre Dame, Notre Dame, IN 46556 USA; Department of Biological Sciences, University of Notre Dame, 100 Galvin Life Sciences Center, Notre Dame, IN 46556 USA

## Abstract

**Background:**

Agent-based models (ABMs) have been used to model the behaviour of individual mosquitoes and other aspects of malaria. In this paper, a conceptual entomological model of the population dynamics of *Anopheles gambiae* and the agent-based implementations derived from it are described. Hypothetical vector control interventions (HVCIs) are implemented to target specific activities in the mosquito life cycle, and their impacts are evaluated.

**Methods:**

The core model is described in terms of the complete *An. gambiae* mosquito life cycle. Primary features include the development and mortality rates in different aquatic and adult stages, the aquatic habitats and oviposition. The density- and age-dependent larval and adult mortality rates (vector senescence) allow the model to capture the age-dependent aspects of the mosquito biology. Details of hypothetical interventions are also described.

**Results:**

Results show that with varying coverage and temperature ranges, the hypothetical interventions targeting the gonotrophic cycle stages produce higher impacts than the rest in reducing the potentially infectious female (PIF) mosquito populations, due to their multi-hour mortality impacts and their applicability at multiple gonotrophic cycles. Thus, these stages may be the most effective points of target for newly developed and novel interventions. A combined HVCI with low coverage can produce additive synergistic impacts and can be more effective than isolated HVCIs with comparatively higher coverages. It is emphasized that although the model described in this paper is designed specifically around the mosquito *An. gambiae*, it could effectively apply to many other major malaria vectors in the world (including the three most efficient nominal anopheline species *An. gambiae*, *Anopheles coluzzii* and *Anopheles arabiensis*) by incorporating a variety of factors (seasonality cycles, rainfall, humidity, etc.). Thus, the model can essentially be treated as a generic *Anopheles* model, offering an excellent framework for such extensions. The utility of the core model has also been demonstrated by several other applications, each of which investigates well-defined biological research questions across a variety of dimensions (including spatial models, insecticide resistance, and sterile insect techniques).

## Background

Modelling can play important roles to quantify the effects of malaria-control interventions and to answer other interesting research questions. Historically, malaria modelling has emerged using two primary methodologies: mathematical and agent-based. Mathematical modelling, being predominantly deterministic and differential equation-based, dates back to the early malaria transmission models of Ross and Macdonald [1, 2]. On the other hand, agent-based models (ABMs), also known as individual-based models, have been used to model the basic behaviour of individual mosquitoes (‘agents’), including interactions between agents and to their local environment. Compared to mathematical models, ABMs offer certain unique features which include the ability to incorporate individual variability of agents and to investigate macroscale properties by integrating microscale interactions [3]. In recent years, with the unprecedented rates of advance in computer technology (in both hardware and software), ABMs have been increasingly adopted by malaria epidemiology researchers (especially modellers). A summary comparing important model features (e.g., spatial representation, time step resolution, interventions modelled, etc.) from some recent malaria models is given in [4, 5].

*Anopheles gambiae* is the major vector of malaria in much of sub-Saharan Africa. Due to its pivotal role in malaria transmission, modelling its population dynamics can assist in finding factors in the mosquito life cycle that can be targeted to decrease malaria. In this paper, a conceptual entomological model (hereafter referred to as the core model) of the population dynamics of *An. gambiae* and the agent-based implementation (hereafter referred to as the *ABM*) derived from the core model are described. The *An. gambiae* complex, a closely related group of eight named mosquito species found primarily in Africa, includes the three nominal species, *An. gambiae*, *Anopheles coluzzii* and *Anopheles arabiensis,* that are among the most efficient malaria vectors known. The core model described in this paper has been designed specifically around the mosquito *An. gambiae*. While the respective ecologies and involvement in malaria transmission among other members of the *An. gambiae* complex differ in important ways, this model could effectively apply to many of the several dozen other major malaria vectors in the world.

The core model addresses several important features of the *An. gambiae* life cycle, including the development and mortality rates in different stages, the aquatic habitats, oviposition, etc. Another important feature, vector senescence, is adopted to account for the age-dependent aspects of the mosquito biology, and implemented using density- and age-dependent larval and adult mortality rates.

Preliminary versions of the models, which mostly dealt with exploratory features, have been previously described elsewhere [6–8]. The version described in this paper reflects the most recent updates in an attempt to enrich the models with features that reflect the population dynamics of *An. gambiae* in a more comprehensive way. A summary of major improvements is given in Table 1.

**Table 1:** **Summary of updated features in the current models**

Feature	Previous versions	Current version
Time step resolution	Daily	Hourly
Host-seeking and oviposition	Anytime	Only at night
Stage transitions	Anytime	Only during permitted time-windows
Egg development time	Constant	Temperature-dependent; consists of egg incubation and hatching times

### Vector control interventions

The last decade (2000–2010) of worldwide malaria control efforts has seen an unprecedented increase in the coverage of malaria control interventions, with insecticide-treated nets (ITNs) or long-lasting impregnated nets (LLINs), indoor residual spraying (IRS), and larval source management (LSM) as front-line vector control tools [9]. Impact of these interventions, often applied in isolation and in combination, has been investigated by numerous studies [10–12]. In addition to the time-tested, established tools such as ITNs, IRS and LSM, new and novel intervention tactics and strategies in the form of new drugs, vaccines, insecticides, improved surveillance methods, etc. are also being investigated [13]. Some of the promising approaches include genetically engineered mosquitoes through sterile insect technique (SIT) or release of insects containing a dominant lethal (RIDL) [14, 15], fungal biopesticides [16], the development of genetically modified mosquitoes (GMMs) or transgenic mosquitoes [17], transmission blocking vaccines (TBVs) [18], etc. Recent studies have also shown the potential of applying current vector control interventions in various transmission settings in an integrated vector management (IVM) approach, promoted by the World Health Organization (WHO) [9].

The ABM provides an excellent framework to target specific activities in the mosquito life cycle and to explore the impacts of vector control interventions that combine multiple strategies to reduce dependence on any single tool. Instead of directly exploring established tactics such as LSM or ITNs, the ABM theoretically implements and compares the effectiveness of various hypothetical vector control interventions (HVCIs). The HVCIs are carefully selected so that they may be mapped to current real-world interventions, and the model explores their application both in isolation and in combination. The utility of the core model is also demonstrated by several other extensions (published elsewhere).

## Methods

### The core model

**Figure 1:**
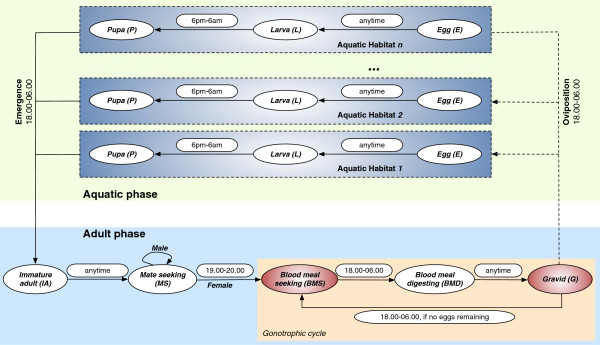
**Life cycle of mosquito agents in the agent-based model.** The *An. gambiae* mosquito life cycle consists of aquatic and adult phases. The aquatic phase consists of three aquatic stages: *Egg (E)*, *Larva (L)*, and *Pupa (P)*. The adult phase consists of five adult stages: *Immature adult (IA)*, *Mate seeking (MS)*, *Blood meal seeking (BMS)*, *Blood meal digesting (BMD)*, and *Gravid (G)*. Each oval represents a stage in the model. Permissible time transition windows from one stage to another are shown next to the corresponding stage transition arrows as rounded rectangles (e.g., 18.00-06.00). Note that adult males, once reaching MS stage, remain forever in that stage until they die; adult females cycle through obtaining blood meals (in BMS stage), developing eggs (in BMD stage), and ovipositing the eggs (in G stage) until they die. The two resource-seeking adult stages (BMS and G) are marked in red. Adapted and updated from [4].

The complete *An. gambiae* mosquito life cycle consists of *aquatic* and *adult* phases, as shown in Figure 1. The aquatic phase consists of three aquatic stages: egg, larva and pupa. The adult phase consists of five adult stages: immature adult, mate seeking, blood meal seeking, blood meal digesting, and gravid. The development and mortality rates in all eight stages of the life cycle are described in terms of the aquatic and adult mosquito populations. All symbols and parameters used in the core model are summarized in Table 2.

**Table 2:** **Symbols and parameters used in the core model**

Parameter	Description	Unit	Default value
*T*	Ambient temperature	°C	30
*P*_*FindHost*_	Probability of a female adult to find a human host	N/A	25%
*P*_*FindBloodMeal*_	Probability of a female adult to find a blood meal	N/A	100%
*P*_*FindHabitat*_	Probability of a female adult to find an aquatic habitat	N/A	25%
*HC*	Habitat capacity	N/A	1000
*r*	Combined seasonality factor	N/A	1.0
*Biomass*	Age-adjusted biomass in a habitat	N/A	Dynamic
*N*_*Eggs*_	Number of eggs in a habitat	N/A	Dynamic
*N*_*e*_	One-day old equivalent larval population	N/A	Dynamic
*N*_*Pupae*_	Number of pupae in a habitat	N/A	Dynamic
*Age*_*Cohort*_	Common age of a cohort	Day	Dynamic
*N*_*LarvaePerCohort*_	Number of larvae in an age-cohort	N/A	Dynamic
*gcn*	Gonotrophic cycle number	N/A	Dynamic
*Eggs*	Maximum number of eggs a female can lay	N/A	Dynamic
*N (170, 30)*	Normal distribution for fecundity in the first gonotrophic cycle	N/A	*mean* =170, *sd* =30
*Eggs*_*Potential*_	Potential number of eggs a female is allowed to lay	N/A	Dynamic
*w*	Habitat sampling weight (within the same gonotrophic cycle)	N/A	1, 2 or 3
DMR	Daily mortality rate	Day^-1^	0.1
HMR	Hourly mortality rate	Hour^-1^	0.00438
α	Baseline DMR (for larvae and adults)	Day^-1^	0.1
β	Exponential mortality increase with age	N/A	0.04
*s*	Degree of mortality deceleration	N/A	0.1

Parameters are listed in order of appearance in the text. *N/A* means not applicable or not available. *Dynamic* means the parameter value can change within a simulation run; *sd* denotes standard deviation.

### The aquatic phase

Since malaria vectors are poikilothermic, the ambient temperature is a critical variable in the growth and development kinetics of *An. gambiae* [19, 20]. Thus, development rates in most stages in the core model are temperature-dependent. The aquatic stages are described below.

#### Egg (E)

**Figure 2:**
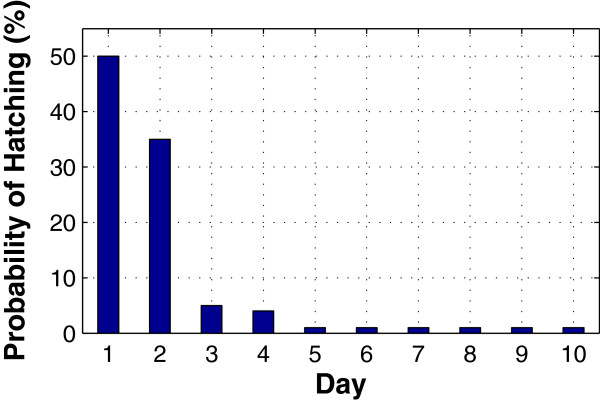
**The egg-hatching time distribution.** The *An. gambiae* egg-hatching time distribution has a strong positive skew, with 89% of the eggs hatching during the second and third day after oviposition, 10% hatching during the next four days, and the remaining 1% hatching over the subsequent week [20]. The x-axis denotes hatching time (in days), and the y-axis denotes the probability of hatching.

Development in the egg stage is comprised of two distinct components: incubation and hatching. Egg incubation depends on the ambient temperature, and usually requires one to two days. It is governed by a linear function:
1

where *T* is the ambient temperature (in °C) in the range 15 ≤ *T* ≤40. As found in a recent study, *An. gambiae* egg-hatching time distribution has a strong positive skew, with 89% of the eggs hatching during the second and third day after oviposition, 10% hatching during the next four days, and the remaining 1% hatching over the subsequent week [21]. The egg-hatching time distribution is shown in Figure 2.

#### Larva (L)

Development in the larva stage primarily depends on temperature. To model the growth and development of larvae, the enzyme kinetics model used in Depinay *et al.* [19] is adopted. Details about the enzyme kinetics model can be found in [22, 23].

The mean larval development rate per hour at temperature *T* (in K), *r(T*_*K*_*)*, is given by Equation 2:
2

where all parameters and their values are summarized in Table 3. Equation 2 is derived from the basic assumption that poikilotherm (*An. gambiae* larvae in this case) development is regulated by a single control enzyme, whose reaction rate determines the development rate of the organism [19]. The mean larval development rate per hour at temperature *T* (in °C), *r(T)*, is obtained by substituting the parameters from Table 3 in Equation 2:
3

**Table 3:** **Larval development parameters for** ***Anopheles gambiae***

Parameter	Description	Unit/dimension	Value used
*ρ*_25*°C*_	Larval development rate per hour at 25°C, assuming that there is no temperature inactivation of the critical enzyme	Hour^-1^	0.037
*R*	The universal gas constant	cal × K^-1^ × mol^-1^	1.987
	The enthalpy (change) of activation of the reaction catalyzed by the enzyme	cal × K^-1^ × mol^-1^	15684
*ΔH*_*L*_	The enthalpy change associated with low temperature inactivation of the enzyme	cal × K^-1^ × mol^-1^	-229902
*ΔH*_*H*_	The enthalpy change associated with high temperature inactivation of the enzyme	cal × K^-1^ × mol^-1^	822285
	The temperature where 50% of the enzyme is inactivated by low temperature	K	286.4
	The temperature where 50% of the enzyme is inactivated by high temperature	K	310.3

The enzyme kinetics model and the values used are adopted from Depinay *et al.* [19]. K represents temperature in Kelvin. *ρ*_25*°C*_ relates the standard reference temperature (25°C) at which most poikilotherms experience little low or high temperature enzyme inactivation. Details about the enzyme kinetics model can be found in [22, 23].

The cumulative larval development, *CD*_*larva*_, is accumulated *per day* through days 0, 1,…*n*:
4

To complete the larval development, a threshold is defined with a normal random variable *N* (to allow for 10% variability); thus, the larval development is completed (i.e., the pupation begins) when:
5

#### Pupa (P)

Development in the pupa stage, which also depends on temperature, is modelled similarly as in *Incubation*_*Egg*_*(T)*, and follows Equation 1.

### The adult phase

The adult stages are described below:

#### Immature adult (IA)

The immature adult stage contains the period (one to three days, depending on temperature) after emergence and before the mosquito is sexually capable of mating. Development in this stage is governed by a linear function of temperature, derived from the adult development curves by Depinay *et al.* [19], as shown in Equation 6:
6

where *T* is the ambient temperature (in °C) in the range 15 ≤ *T* ≤40. Substituting *T* for 36°C, 27°C, and 18°C in Equation 6 yields the corresponding development times of one, two and three days, respectively.

#### Mate seeking (MS)

The mate seeking stage is assumed instantaneous, and occurs during the first hour of evening (before 19.00, see Figure 1). After entering this stage from the immature adult stage, female adults are assumed to always find males for mating. Male adults stay in the stage for the rest of their lives, while female adults enter the blood meal seeking stage at 19.00 hours. This fixed-duration period is designed to capture various swarming and mating behaviour of anophelines, during which predators and interventions (e.g., outdoor spraying, genetically engineered mosquitoes through SIT, etc.) may cause additional mortality (to be modelled in the future).

#### Blood meal seeking (BMS)

In the blood meal seeking stage, female adults search for blood meals. Since *An. gambiae* has been observed to bite mostly throughout the night [24–27], the host seeking activities are restricted within the time-window of 18.00 to 06.00 (i.e., from dusk to dawn). During each (hourly) time step, the probability of a female adult to encounter a human host, *P*_*FindHost*_, is set as 25% (and remains the same during each hour in the time-window of 18.00 to 06.00). Thus, the blood meal seeking activity does not diminish through the night. Once it finds a host, obtaining a blood meal is always assumed successful (i.e., *P*_*FindBloodMeal*_ is set as 100%), and it immediately enters the blood meal digesting stage (assuming that no interventions are in use). If it cannot find a host, BMS continues to the next time step, and so on, as long as the time-window (18.00-06.00) permits.

#### Blood meal digesting (BMD)

Development in the blood meal digesting stage is also governed by a linear function of temperature, derived from the adult development curves by Depinay *et al.* [19], as shown in Equation 7:
7

where *T* is the ambient temperature (in °C) in the range 15 ≤ *T* ≤40. Depending on the temperature, Equation 7 yields the corresponding development times of one to 2.5 days.

#### Gravid (G)

In the gravid stage, a female mosquito lays its developed eggs in batches. Since *An. gambiae* mosquitoes are nocturnal in their oviposition activities [28, 29], oviposition, like host seeking, is also restricted within the time-window of 18.00 to 06.00 in the model. During each (hourly) time step, the probability of a female adult to sample a randomly selected aquatic habitat, *P*_*FindHabitat*_, is set as 25% (and remains the same during each hour in the time-window of 18.00 to 06.00). If all eggs are not laid within the night, it rests until the next night to lay the remaining eggs. Once all the eggs are laid, and the time-window (18.00-06.00) permits, it enters the BMS stage to search for another blood meal, thus starting a new gonotrophic cycle.

### Aquatic habitats

The core model assumes simplistic, homogeneous aquatic habitats (breeding sites). All habitats are uniform in size and capacity, and the water temperature of the habitats is assumed the same as the air temperature. To account for the combined seasonality factor, *r*, each aquatic habitat is set with a habitat capacity (*HC*) as a linear function of *r*:
8

where *HC*_*Baseline*_ represents the baseline habitat capacity. *r* ∈ [0, 1) indicates capacity below the baseline (which may occur due to several seasonality/weather factors, e.g., low precipitation), and r >1 indicates capacity above the baseline (which may occur due to high precipitation, lack of sunlight, etc.). *HC* essentially represents the density-dependent oviposition mechanism by regulating an age-adjusted biomass (see Oviposition) that the habitat can sustain.

### Oviposition

The oviposition behaviour of *An. gambiae* gravid mosquitoes can be affected by a variety of factors, as demonstrated by several studies [30–35]. For example, Koenraadt and Takken [32] showed that *An. gambiae s.l.* females tend to avoid oviposition sites containing older instar larvae, and thus reduce the risk of predation of offspring. Munga *et al.* [33] compared oviposition choices of *An. gambiae* in rainwater conditioned with different numbers and densities of con-specific larvae, and found that in the presence of different densities of larvae, more eggs were laid in rainwater that had the fewest or no larvae; additionally, the greatest number of eggs were laid in rainwater that contained the lowest concentration of larvae. Other studies also showed that increased competition within the larval environment may have negative impacts on several aspects of the life cycle, including larval development rate, adult survivorship, adult body size, fecundity [31, 34].

In the core model, all larvae are sorted into different age groups, or cohorts, according to the *common age* of the cohort. The model keeps track of the age-adjusted biomass, Biomass, in each aquatic habitat, which is defined as the sum of the eggs, pupae, and the one-day old equivalent larval population, *N*_*e*_ (see Equation 10), in the habitat:
9

where *N*_*Eggs*_ and *N*_*Pupae*_ represent the number of eggs and pupae, respectively, in the selected habitat. *N*_*e*_ is computed by first multiplying the number of larvae in each age-cohort (*N*_*LarvaePerCohort*_) by the cohort’s age (*Age*_*Cohort*_, in days), and then summing up the values for all age-cohorts:
10

This, in turn, provides a *check and balance* mechanism for the model, since for each habitat, the habitat capacity *HC* may serve as a soft upper limit for the aquatic population (by limiting the larval density and biomass of the habitat).

Since *An. gambiae* females have been reported to exhibit skip-oviposition (using several different habitats to oviposit a few eggs in each) [36], the core model assumes that a gravid female lays the developed eggs in multiple habitats (within the time-window of 18.00 to 06.00), and its inclination to lay the remaining eggs successively increases as it visits more habitats. Due to several factors, *An. gambiae* fecundity can be affected to produce smaller egg-batch sizes [11, 37]. Thus, in successive gonotrophic cycles, the maximum number of eggs a female can lay, *Eggs*_*Max*_*(gcn)*, is reduced by 20% from the previous cycle:
11

where *gcn* represents the gonotrophic cycle number, and *N(170, 30)* represents the fecundity in the very first gonotrophic cycle, drawn from a normal distribution with *mean* =170 and *standard deviation* =30.

Successive oviposition attempts within the *same* gonotrophic cycle are distinguished by a habitat sampling weight, *w*. To account for the composite factors that arise from conspecific density and competition, the age-adjusted biomass (Biomass) is checked against the *HC* to determine the potential number of eggs, *Eggs*_*Potential*_*(gcn, w)*, that a female is allowed to lay in a given habitat:
12

Once all the eggs are laid, the current gonotrophic cycle is completed, and the gravid female starts a new cycle by entering into the BMS stage (within the time-window of 18.00 to 06.00).

### Mortality rates

In most epidemiology models, the mortality of the organisms being modelled plays a crucial role in shaping the model’s characteristics. Daily mortality rate is the most important determinant of a mosquito’s ability to transmit pathogens [38]. Traditionally, most malaria transmission models assume age-independent (i.e., non-senescent) vector mortality. However, non-senescence of the vector could only lead to approximate estimates and misleading predictions since they obscure the age-dependent aspects of the mosquito biology. It assumes an unrealistic, simplified view that the vector potential of all mosquitoes, regardless of their age, is the same. This, in turn, also affects other determinants of pathogen transmission (e.g., biting rate, host preference, vector competence, dispersal, resistance to insecticides).

Several studies have shown the impact of vector senescence on malaria transmission. The longer a mosquito lives, the less likely it is to survive if the mosquito senesces. Thus, small changes in daily mortality can result in relatively large changes in the pathogen transmission cycle. For example, using large-scale laboratory life-table techniques, Styer *et al.* showed that mosquito mortality was low at young age (less than ten days old), steadily increased at middle age, and decelerated at older age [38]. Clements and Paterson showed that the mortality and survival rates in most wild populations of mosquitoes of 11 tropical species are age-dependent [39]. Bellan showed that the classical constant (age-independent) mortality rate led most transmission models to overestimate the effectiveness of interventions which reduce the mosquito survival rate, and concluded that future transmission models that examine antivectorial interventions should incorporate realistic age-dependent mortality rates [40]. Based on the above observations, age-specific mortality rates are used for the larva stage, and for all the adult stages in the core model.

### Aquatic mortality rates

Since in general, *An. gambiae* egg and pupa survival are not density-dependent, the daily mortality rates of egg and pupa stages are set as an empirical constant of 10%, yielding the hourly mortality rate of 0.00438:
13

Mortality of larvae is affected by a variety of factors, which include the age of larvae [11, 19, 39, 41], the density-dependent effects arising from predation, cannibalism and resource competition in the larval population [19, 32], habitat capacity, and weather factors such as rainfall [11, 19, 42]. The daily mortality rate (DMR) for the larva stage is computed on a per-cohort basis using the cohort’s age *Age*_*Cohort*_ (in days), the age-adjusted one-day old equivalent larval population *N*_*e*_ (see Equation 10), and the habitat capacity *HC*:


where α represents the baseline DMR (set as an empirical constant of 10%), and *HMR*_*Larva*_*(Age*_*Cohort*_*)* represents the corresponding hourly mortality rate (HMR).

### Adult mortality rates

For the adult stages, DMRs are calculated by using a modified version of the logistic mortality model, in which the age-dependent component of mortality increases exponentially with age [38]. Newly emergent adults have a baseline DMR of α. However, as they age, the age-specific mortality rate for each age-cohort is calculated as:


where α is the baseline DMR (10%), β is the exponential mortality increase with age (0.04), *s* is the degree of mortality deceleration (0.1), and *Age*_*Cohort*_ is the common age of the cohort. Note that while many of the coefficients and parameters described above are specified as constant values (e.g., *P*_*FindHost*_ =25%), these values can be calibrated (tuned) to reflect specific scenarios as needed. The theoretical application of the interventions is described next.

### Hypothetical vector control interventions (HVCIs)

Most of the stages of the mosquito life cycle can be thought of as collections of specific activities. Some of these activities are common, i.e., may occur in several life cycle stages. The core model implements HVCIs based on these common activities, which include Entering, Updating, Foraging, and Resting.

*Entering* targets a female mosquito agent when it enters a life cycle stage, essentially working as a one-time hazard, which means that if the mosquito can pass this one-time hazard, it can survive the rest of the stage. *Updating* imposes additional mortality in each hourly time step of the targeted stage, and continues as long as the mosquito remains in the stage; thus, the impact of elevated mortality on mosquitoes imposed by these HVCIs can last through the whole stage. *Foraging* targets female mosquitoes during their resource-seeking activities (host seeking to obtain blood meals and searching for aquatic habitats to oviposit), and continues in each hourly time step as long as the mosquito is seeking resources; lastly, *Resting* acts during the resting (being ‘inactive’) period of the targeted stage, and continues in each hourly time step as long as the mosquito is resting. Note, however, that an assigned killing parameter value (see below) is apportioned over the complete number of hourly time steps in the stage such that the total stage interval is subject to the killing parameter value.

The durations of activities (Entering, Updating, Foraging, and Resting) in the corresponding life cycle stages of different HVCIs are expected to influence the overall qualitative impacts of the HVCIs: the Entering HVCI type imposes a one-time killing effect applied in a single time step (hour), whereas the other three HVCI types (Updating, Foraging, and Resting) impose additional, continuous killing effects that may sustain every hour during the entire stage or multiple consecutive hours in the stage.

The first element of an HVCI denotes the life cycle stage (or in some cases, multiple stages, which are enclosed in parentheses). The second element denotes the activity of the mosquito agent in the corresponding stage(s) and appears as a subscript. The HVCIs are described below:

- ***L***_***Entering***_**:** imposes a one-time killing effect when an egg enters the larva stage. Thus, it simulates a one-time hazard on an egg, essentially simulating LSM by insecticidal control;
- ***L***_***Updating***_**:** imposes an additional killing effect for a larva, and the killing effect sustains every hour (during the entire larval development stage; see Equations 2, 3, 4 and 5) until the larva enters the pupa stage or dies. Thus, *L*_*Updating*_ simulates a continuous killing effect on a larva, essentially simulating LSM by means of biological control*;*
- ***IA***_***Resting***_**:** works similarly as *L*_*Updating*_, imposing an additional killing effect for a mosquito during every hour in the immature adult stage;
- ***BMS***_***Foraging***_**:** imposes an additional killing effect for a host seeking female mosquito in the blood meal seeking stage. The killing effect is in action whenever a female encounters a host (with *P*_*FindHost*_ =25%, meaning 25% probability of finding a host in each hour during BMS), and continues every hour until the mosquito successfully gets a blood meal (and then enters the blood meal digesting stage) or dies; if the female fails to find a host and still survives, it will have to wait in the BMS stage until the next time step, and then repeat the host seeking process. Thus, *BMS*_*Foraging*_ simulates a continuous killing effect on a host seeking female during the entire BMS stage, essentially simulating ITNs and LLINs;
- ***BMS***_***Foraging, K=0%***_**:** being a special case, it is similar to *BMS*_*Foraging*_, with the exception that it has no killing effectiveness. Thus, it simulates a continuous effect (with no additional killing) on a host seeking female during the entire BMS stage, essentially simulating untreated bed nets;
- ***BMD***_***Resting***_**:** imposes an additional killing effect for a female mosquito in the BMD stage, and the killing effect sustains every hour (of the one to 2.5 days duration in BMD; see Equation 7) until the mosquito enters the gravid stage or dies. Thus, *BMD*_*Resting*_ simulates a continuous killing effect on a female mosquito during the entire BMD stage, essentially simulating IRS;
- ***G***_***Foraging***_**:** works similarly as *BMS*_*Foraging*_, imposing an additional killing effect for a female mosquito in the G stage seeking an aquatic habitat for oviposition. The killing effect is in action whenever a female is seeking a habitat (with *P*_*FindHabitat*_ =25%, meaning 25% probability of finding a habitat in each hour during G), and continues every hour until it successfully finds a habitat or dies. Since the core model implements skip-oviposition (using several different habitats to oviposit a few eggs in each), the duration of the killing effect increases with each successive oviposition attempt. Thus, *G*_*Foraging*_ simulates a continuous killing effect on a habitat-seeking female during the entire G stage, essentially simulating lethal ovitraps.

Some HVCIs also target multiple life cycle stages. For example, *(IA, BMS, BMD, G)*_*Entering*_ targets the four stages IA, BMS, BMD and G, imposing increased mortality for mosquitoes entering these stages. It resembles the more recent form of mosquito control using ATSB where the mosquito is essentially seeking a sugar meal each day. ATSB kills female and male mosquitoes searching for essential sugar sources in the outdoor environment [43]. Using an *attract-and-kill* principle, it uses fruit or flower scent as an attractant, sugar solution as a feeding stimulant, and oral toxin to kill the mosquitoes [44].

Intervention parameters (coverage and killing) are denoted as the third element of a corresponding HVCI. Coverage *C* is defined as the probability of encountering an HVCI in a particular stage. Killing *K*, which refers to an increased mortality, toxicity, or killing efficiency (e.g., due to insecticidal effects), is defined as an increased probability of death of a mosquito: for Entering, *K* is defined as the probability with which a mosquito will be killed after it enters the corresponding stage; and for the other three types (Updating, Foraging, and Resting), *K* is defined as an additional, continuous mortality that may occur over multiple consecutive hours in the corresponding stage. Since the core model implements hourly time steps, the latter can be interpreted as an additional hourly mortality of . For example, during the updating activity in a stage, *K* =75% imposes an additional hourly mortality of , or 0.056, for the mosquito agent. Out of numerous possible choices that can be generated by combining different life cycle stages with the activities, the HVCIs are carefully selected so that they may be mapped to current real-world interventions, as described in Table 4.

**Table 4:** **Hypothetical vector control interventions**

HVCI	Interpretation	Real-world mapping
*L*_*Entering*_	Imposes a one-time killing effect (a one-time hazard) when an egg enters the larva stage	LSM by insecticidal control
*L*_*Updating*_	Imposes an additional killing effect for a larva; the killing effect sustains every hour during the entire larval development stage (see Equations 2–5) until the larva enters the pupa stage or dies	LSM by biological control
*BMS*_*Foraging*_	Imposes an additional killing effect for a host seeking female mosquito in the BMS stage; the killing effect is in action whenever a female encounters a host (with *P*_*FindHost*_ =25%) in BMS, and occurs over every hour until the mosquito successfully gets a blood meal (and then enters the BMD stage) or dies; may occur in multiple gonotrophic cycles	ITNs
*BMS*_*Foraging, K=0%*_	A special case of *BMS*_*Foraging*_ with no killing effectiveness; simulates a continuous effect (with no additional killing) on a host seeking female during the entire BMS stage; may occur in multiple gonotrophic cycles	Untreated bed nets
*BMD*_*Resting*_	Imposes an additional killing effect for a female mosquito in the BMD stage, and the killing effect sustains every hour of the 1–2.5 days duration in BMD (see Equation 7) until the mosquito enters the G stage or dies; may occur in multiple gonotrophic cycles	IRS
*G*_*Foraging*_	Imposes an additional killing effect for a female mosquito in the G stage seeking an aquatic habitat for oviposition; the killing effect is in action whenever a female is seeking a habitat (with *P*_*FindHabitat*_ =25%) in G, and occurs over every hour until it successfully finds a habitat or dies; may occur in multiple gonotrophic cycles	Lethal ovitraps
*(IA, BMS, BMD, G)*_*Entering*_	Imposes an additional killing effect (a one-time hazard) for female mosquitoes entering the IA, BMS, BMD, and G stages; may occur in multiple gonotrophic cycles during BMS, BMD, G	ATSB
*(IA, BMS, BMD, G)*_*Resting*_	Imposes an additional killing effect for female mosquitoes resting in the IA, BMS, BMD, and G stages; the killing effect occurs over every hour (when mosquitoes are resting) in all of these stages, and in multiple gonotrophic cycles during BMS, BMD, G	Sugar meal traps

The first element of an HVCI denotes the life cycle stage (or in some cases, multiple stages, which are enclosed in parentheses). The second element denotes the activity of the mosquito agent in the corresponding stage(s) and appears as a subscript. Optionally, a third element may denote the intervention parameters of coverage (*C*) and/or killing (*K*). For each HVCI, its interpretation by the model is described in column 2, and possible mapping to its real-world counterpart is listed in column 3.

The above formulation allows the core model to generate versatile HVCI scenarios that resemble existing real-world interventions. For example, *BMS*_*Foraging, K=0%*_ represents untreated bed nets, the role and importance of which have been shown by many recent studies [45, 46]. Untreated nets, which may not directly increase the mortality of host-seeking females, may still provide some protection and achieve community-wide effects where malaria transmission is reduced when most people in the population regularly sleep under nets [46]. It also allows the model to examine the impact of an HVCI arising from subtle implementation details, and to select from multiple implementation strategies based on these details. For example, the HVCI *G*_*Foraging*_ (which targets female mosquitoes that are searching for aquatic habitats) may be implemented in two ways: (1) an affected female is not killed by the HVCI, but is forced to search for other habitats to lay the remaining eggs, or (2) it is allowed to lay the eggs, but the eggs are not commissioned into the system (i.e., are thrown away). The latter corresponds to lethal ovitraps, which are used as a promising new tool for monitoring and control of the dengue vector *Aedes aegypti* [47]. However, simulation results of these strategies do not show any significant differences, and the second strategy is adopted by the core model.

### Output indices

Female adult mosquito abundance and potentially infectious female *(PIF)* abundance are treated as the primary outputs of the model. PIF denotes the number of female adult mosquitoes that are potentially capable of transmitting malaria. A female adult becomes potentially infectious after it successfully obtains a blood meal (thus presumably has an opportunity to acquire the *Plasmodium falciparum* parasite) and then survives long enough for the parasite to develop and migrate into its salivary gland so that it can be transferred during a subsequent blood meal. Thus, the time period required for a female to become a PIF is greater than or equal to the extrinsic incubation period of malaria. The PIF index, which may not fully capture the malaria transmission dynamics (since neither humans nor the parasites are explicitly modelled), should be treated as an approximate index of the mosquito population that possess the potential to transmit malaria. In reporting the one-year simulation results, the first 100 days, which constitute the warm-up period necessary to reach a steady state, are always omitted for brevity.

### Model assumptions

The current work is theoretical. The presence of only one vector, *An. gambiae*, is assumed. The vector life cycle dynamics is emphasized, and the parasite life cycle and the malaria transmission cycle are not yet included. Mosquitoes senesce, and their probability of death increases with age. The influence of habitat size, surface area, solar insulation, and other related factors, which may influence habitat capacity, are not modelled explicitly. Seasonality and other weather/climate parameters (except daily temperature) are not included. In aquatic habitats, survival of eggs and pupae is not influenced by the aquatic mosquito density (hence, the mortality rates in the egg and pupa stages are treated as constants). Female adults, once entering the mate seeking stage, are always assumed to successfully find males to mate with (in the next time step). For egg maturation, a single blood meal is assumed sufficient. The mortality rate of female adults is assumed independent of their malaria infectivity states. Female fecundity is assumed normally distributed with mean of 170 and standard deviation of 30.

For HVCIs, coverage entails distribution and usage (i.e., these parameters are not differentiated). HVCIs ignore other related factors such as excitorepellency, and assume the parameters to be uniform over time. HVCIs are applied after the simulation warm-up period, and continued up to the end of the simulation. In figures that represent abundance plots, each colour-coded plot represents a specific value of a parameter (e.g., coverage *C*), with colour keys presented in the legend.

### Simulations

The model is implemented as computer simulations in both Java and C++ (to compare language-specific dependencies as well as verification and validation features). The default time step used in the simulations is one hour. Each simulation starts with a prespecified initial number of eggs as agents, typically consisting of a 50:50 male–female ratio. Other input parameters include prespecified numbers of habitats with adjustable habitat capacities, hourly temperature profiles, habitat capacity profiles, etc. During parameter sweep experiments, each simulation is repeated at least 30 times to eliminate any biases introduced by different sources of randomness (stochasticity), the behaviour uncertainties of the agents’ actions, states, etc. With around 25,000 adults and one million aquatic mosquitoes in the system, a sample simulation run takes about an hour to output one-year results data on a 2.4 GHz Intel Core 2 Duo computer with 2 GB of memory.

## Results

In the following, the results of various HVCIs are described.

### Impact of varying coverage (*C*)

**Figure 3:**
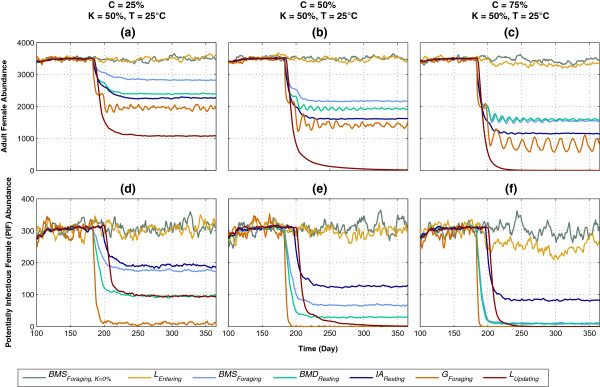
**Impact of varying coverage (** ***C*** **) on abundance and potentially infectious female with hypothetical vector control interventions. (a)-(c)** depict abundance, and **(d)-(f)** depict PIF. Each column represents a specific coverage (*C*) for HVCIs (e.g., *C* =50%). The legend at the bottom shows the HVCIs modelled. Each colour-coded plot represents a specific HVCI, with colour keys presented in the legend. The x-axis denotes simulation time (in days), and the y-axis denotes abundance or PIF. HVCIs are applied on day 100, and continued up to the end of the simulation. The first 100 days, which constitute the warm-up period necessary to reach a steady state, is omitted from the one-year simulation results.

The impact of varying coverage (*C*) on mosquito abundance and PIF abundance of seven HVCIs, applied in isolation in five stages (L, IA, BMS, BMD, and G) of the mosquito life cycle, is shown in Figure 3. Three levels of *C* are simulated: low (*C* =25%), moderate (*C* =50%), and high (*C* =75%), with killing *K* and ambient temperature *T* being fixed at 50% (except for the special case of *BMS*_*Foraging, K=0%*_) and 25°C, respectively. Figures 3a-c show adult female abundance. As shown in Figure 3a with low (*C* =25%) coverage, *L*_*Updating*_ performs the best in reducing abundance by ≈ 72%, and is followed by *G*_*Foraging*_, *IA*_*Resting*_, *BMD*_*Resting*_, and *BMS*_*Foraging*_. As *C* is increased to 50 and 75% (Figure 3b and c), *L*_*Updating*_ reduces abundance to zero and essentially eliminates the adult mosquito populations, and the differences in impact between *BMS*_*Foraging*_ and *BMD*_*Resting*_ are gradually diminished. Both *L*_*Updating*_ and *G*_*Foraging*_ perform better than other interventions due to the fact that they impose additional killing effects for much longer durations: the impact of *L*_*Updating*_ occurs over *every hour* in the entire larval development stage (which is the longest life cycle stage), and the impact of *G*_*Foraging*_, which occurs over every hour during the habitat-seeking period, is also further increased by: 1) multiple oviposition attempts, i.e., skip-oviposition, and 2) being applied multiple times in multiple gonotrophic cycles (see below). However, for any coverage level, *BMS*_*Foraging, K=0%*_ and *L*_*Entering*_ produce no visible impacts, as can be seen in Figures 3a-c. This is not surprising, because *BMS*_*Foraging, K=0%*_ imposes no additional mortality on the host seeking female, and *L*_*Entering*_ is applied (to a larva) for only a single hourly time step.

Figures 3d-f, which show PIF abundance, depict significantly different patterns of impact for the HVCIs. With all coverage levels, except for the two weaker HVCIs *BMS*_*Foraging, K=0%*_ and *L*_*Entering*_ (weaker as explained above), the other five are able to reduce PIF abundance significantly. Importantly, *G*_*Foraging*_ is the most effective HVCI at all coverage levels, followed by *L*_*Updating*_ and *BMD*_*Resting*_. With *C* =25%, *G*_*Foraging*_ reduces PIF abundance to almost zero, essentially eliminating the malaria transmission capacity of the mosquito population (although the adult population still survives). The higher impacts of *G*_*Foraging*_ and *BMD*_*Resting*_ are a consequence of the fact that these HVCIs include both multi-hour mortality applications as well as impacts during multiple gonotrophic cycles. However, the impact of *L*_*Updating*_, although being applied only once in the mosquito’s life cycle, also improves as *C* increases, as a consequence of having the longest duration (thus being applied for comparatively more number of time steps). Apart from *G*_*Foraging*_ and *BMD*_*Resting*_, the other HVCI that also targets the adult females during the gonotrophic cycle stages, *BMS*_*Foraging*_, also produce significant impact (especially with *C* =75%).

This points to an important insight: in general, progressively higher impacts are observed with the three HVCIs as the life cycle stages advance from BMS to BMD, and finally G (resembling ITNs, IRS, and lethal ovitraps (see Table 4), respectively). Since they target the gonotrophic cycle stages (and thus are applied multiple times in multiple gonotrophic cycles, affecting higher proportions of the progressively older female mosquitoes), they are able to impose additional killing effects for much longer durations. As a result, they perform progressively better – befitting the definition of PIF abundance which captures the older mosquito populations (that survive long enough to possess the potential to transmit malaria).

Interestingly, *L*_*Updating*_ and *IA*_*Resting*_, which do not target the gonotrophic cycle stages, depict the presence of a time lag (a short duration of roughly 20 days) before the PIF abundances significantly go down, possibly indicating the time delay necessary to propagate the impacts of these HVCIs on the older mosquito populations, and the fact that they are applied only once in the mosquito’s life cycle.

### Impact of varying temperature (*T*)

**Figure 4:**
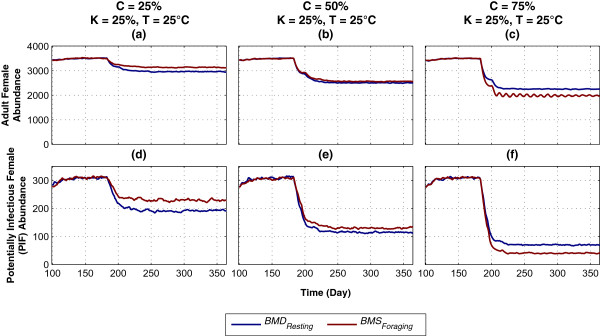
**Impact of varying coverage (** ***C*** **) with low killing (** ***K*** **=25%) on abundance and potentially infectious female, with hypothetical vector control interventions** ***BMS***_***Foraging***_ **and** ***BMD***_***Resting***_ **. (a)-(c)** and **(d)-(f)** depict abundance and PIF, respectively. Killing *K* is fixed at a low level of 25%. Each column represents a specific coverage (*C*) for HVCIs (e.g., *C* = 50%). Each colour-coded plot represents a specific HVCI, with colour keys presented in the legend. The x-axis denotes simulation time (in days), and the y-axis denotes abundance or PIF. HVCIs are applied on day 100, and continued up to the end of the simulation. The warm-up period is omitted from the results.

The impact of varying the ambient temperature (*T*) on abundance and PIF of the same seven HVCIs is shown in Figure 4. Four levels of *T* are simulated: 22°C, 25°C, 30°C, and 36°C, with coverage *C* and killing *K* both being fixed at moderate levels of 50% (except for the special case of *BMS*_*Foraging, K=0%*_). Figures 4a-d show adult female abundance. In the low-mid temperature range (22-25°C), *L*_*Updating*_ performs the best, reducing female abundance to zero, and is followed by *G*_*Foraging*_, *IA*_*Resting*_, *BMD*_*Resting*_, and *BMS*_*Foraging*_. In the mid-high temperature range (30-36°C), both *L*_*Updating*_ and *G*_*Foraging*_ perform significantly better than the other interventions due to the additional killing effects being applied for much longer durations: *L*_*Updating*_ occurring over *every hour* in the entire larval development stage, and *G*_*Foraging*_ occurring over every hour during the habitat-seeking period, which is further increased by skip-oviposition and being applied in *multiple* gonotrophic cycles (as explained above for Figure 3). However, in this range (30-36°C), the impact of *L*_*Updating*_ is diminished: since larval development primarily depends on the temperature (see Equations 2, 3, 4 and 5 in Methods), increasing the temperature accelerates the rate of larval development, effectively shortening the duration of *L*_*Updating*_. For any temperature range, *BMS*_*Foraging, K=0%*_ and *L*_*Entering*_ produce no visible impacts (due to no additional mortality and very short duration, respectively).

Figures 4a-b also show that in the low-mid temperature range (22-25°C), *IA*_*Resting*_ is more effective than *BMD*_*Resting*_ on abundance. However, with *T* ≥30°C, the opposite trends are observed (i.e., *BMD*_*Resting*_ becomes more effective), as shown in Figures 4c-d. These differences are attributed to the temperature-dependent development functions for IA and BMD, as were shown in Equations 6 and 7.

Figures 4e-h show the PIF abundance. In all temperature ranges, *G*_*Foraging*_ performs the best by reducing PIF to ≈ zero, and is followed by *BMD*_*Resting*_, *L*_*Updating*_, and *BMS*_*Foraging*_, while *BMS*_*Foraging, K=0%*_ and *L*_*Entering*_ produce no visible impacts (for the same reasons as explained above for Figure 3). The higher impacts of the three HVCIs *BMS*_*Foraging*_, *BMD*_*Resting*_, and *G*_*Foraging*_, which target the gonotrophic cycle stages (and thus are applied *multiple* times in *multiple* gonotrophic cycles), can be seen in the low-mid temperature range (22-25°C). However, in the mid-high temperature range (30-36°C), the impacts of *IA*_*Resting*_, *BMD*_*Resting*_, and *BMS*_*Foraging*_ are gradually diminished (as was shown in Figure 3 with varying coverage *C*). This is due to the fact that in higher temperatures (i.e., in warm or hot weathers), developments in some of the adult stages happen must faster (see Equations 6 and 7 in Methods). Thus, the durations of these HVCIs are shortened, and their impacts are reduced.

In higher temperatures, the shortened durations of some of the temperature-dependent stages (L, IA, and BMD) also affect the overall mosquito populations (both female abundances and PIFs), as evident from the differences in magnitudes along y-axes in Figure 4 (before the interventions are applied): in Figures 4a-d, mean abundances increase from ≈ 1,600 to ≈ 23,000, and in Figures 4e-h, mean PIFs increase from ≈ 40 to ≈ 8,000.

### Impact of *BMS*_*Foraging*_and *BMD*_*Resting*_with low killing (*K*)

**Figure 5:**
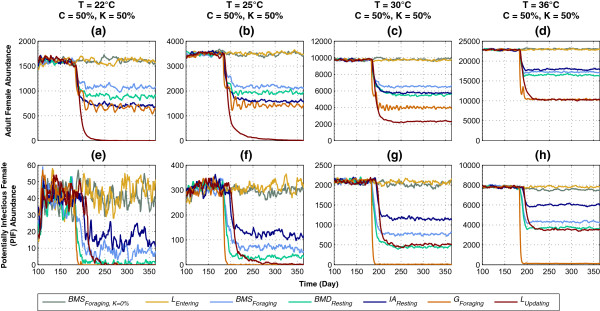
**Impact of varying temperature (** ***T*** **) on abundance and potentially infectious female with hypothetical vector control interventions. (a)-(d)** and **(e)-(h)** depict abundance and PIF, respectively. Four levels of *T* are simulated: 22°C, 25°C, 30°C, and 36°C, with coverage *C* and killing *K* both being fixed at a moderate level of 50% (except for the special case of *BMS*_*Foraging, K=0%*_). Each column represents a specific temperature (e.g., *T* = 25°C). Each colour-coded plot represents a specific HVCI, with colour keys presented in the legend. The x-axis denotes simulation time (in days), and the y-axis denotes abundance or PIF. HVCIs are applied on day 100, and continued up to the end of the simulation. The warm-up period is omitted from the results. Note that the scales on the y-axes are different, reflecting the corresponding mosquito populations obtained by using varying temperatures.

As mentioned before, with killing *K* being fixed at a medium level (50%), the differences in impact between *BMS*_*Foraging*_ and *BMD*_*Resting*_ are gradually diminished as coverage *C* increases (see Figure 3 above). Figure 5 shows the impact of low killing (*K* =25%) on *BMS*_*Foraging*_ and *BMD*_*Resting*_ with varying coverages (25, 50 and 75%). Temperature *T* is being fixed at 25°C. With low-mid coverages (25-50%), *BMD*_*Resting*_ performs better than *BMS*_*Foraging*_ for both abundances (Figure 5a and b) and PIFs (Figure 5d and e). However, with high coverage and low killing, *BMS*_*Foraging*_ performs better than *BMD*_*Resting*_ (Figure 5c and f).

### HVCIs targeting multiple life cycle stages

**Figure 6:**
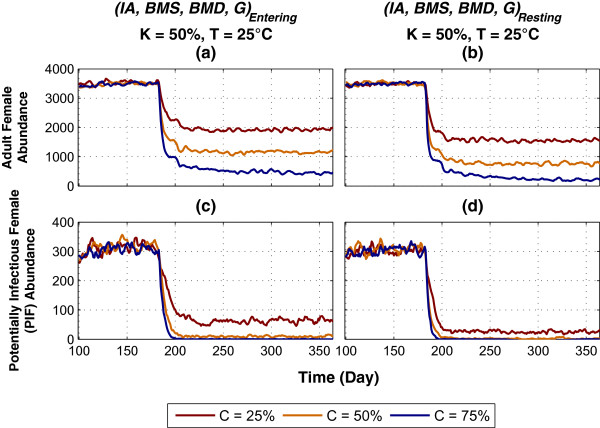
**Impact of varying coverage (** ***C*** **) on abundance and potentially infectious females, with hypothetical vector control interventions applied to multiple life cycle stages (IA, BMS, BMD, G). (a)-(b)** and **(c)-(d)** depict abundance and PIF, respectively. **(a)** and **(c)** represent results for *(IA, BMS, BMD, G)*_*Entering*_; **(b)** and **(d)** represent results for *(IA, BMS, BMD, G)*_*Resting*_. Each colour-coded plot represents a specific coverage (*C*), with colour keys presented in the legend. Killing *K* and temperature (*T*) are fixed at 50% and 25°C, respectively. The x-axis denotes simulation time (in days), and the y-axis denotes abundance or PIF. HVCIs are applied on day 100, and continued up to the end of the simulation. The warm-up period is omitted from the results.

The impact of two HVCIs targeting multiple adult life cycle stages with varying coverage is shown in Figure 6. Note that these HVCIs essentially represent sugar meal traps (that assumes a daily sugar meal by female mosquitoes). Figure 6a and c represent results for *(IA, BMS, BMD, G)*_*Entering*_, and Figure 6b and d represent results for *(IA, BMS, BMD, G)*_*Resting*_. Killing *K* and temperature *T* are fixed at 50% and 25°C, respectively. As shown in Figure 6a and c, with moderate levels (50%) of coverage and killing, *(IA, BMS, BMD, G)*_*Entering*_ can produce considerable impacts on both abundance and PIF, and performs better than *BMS*_*Foraging*_ and *BMD*_*Resting*_ (compare to Figure 3b and e). However, as the coverage rises up to 75%, the PIF populations die off, as shown in Figure 6c. Although *(IA, BMS, BMD, G)*_*Entering*_ places a one-time hazard in each of the four adult stages, it can eliminate the PIF population, because the last three stages (BMS, BMD, G) are the gonotrophic cycle stages, implying impacts during each stage occurring over multiple gonotrophic cycles. Another configuration of *C* =50%, *K* =75%, having the same products of the two parameters, produce similar impact on the outputs.

As shown in Figures 6b and d, the other HVCI, *(IA, BMS, BMD, G)*_*Resting*_, attacking female mosquitoes which are resting, performs better than *(IA, BMS, BMD, G)*_*Entering*_. It kills the PIF population with a moderate coverage level (*C* =50%). This is not surprising, considering the fact that in each stage it imposes both multi-hour mortality applications for much longer durations, as well as impacts during each of the last three stages (BMS, BMD, G) occurring over multiple gonotrophic cycles (also described above in the results of Figure 3).

### Combining multiple HVCIs

**Figure 7:**
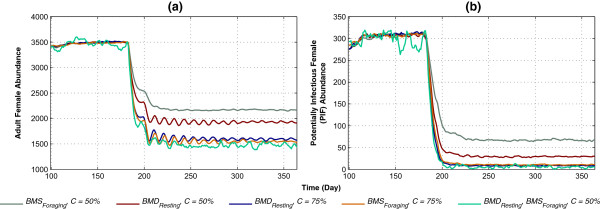
**Impact of** ***BMS***_***Foraging***_ **and** ***BMD***_***Resting***_ **on abundance and potentially infectious female, applied in isolation and in combination. (a)** and **(b)** depict abundance and PIF, respectively. Both HVCIs are applied in isolation with *C* =50% and *C* =75%, and in combination with *C* =50% each, with killing *K* being fixed at 50%. Each colour-coded plot represents a specific case of isolated or combined application, with colour keys presented in the legend. The x-axis denotes simulation time (in days), and the y-axis denotes abundance or PIF. The warm-up period is omitted from the results.

**Figure 8:**
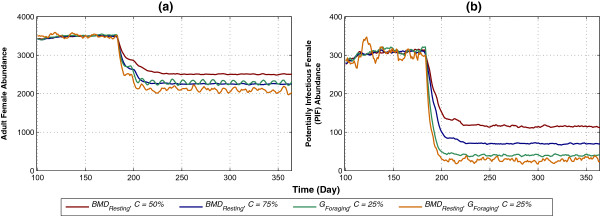
**Impact of** ***BMD***_***Resting***_ **and** ***G***_***Foraging***_ **on abundance and potentially infectious female, applied in isolation and in combination. (a)** and **(b)** depict abundance and PIF, respectively. *BMD*_*Resting*_, with moderate and high levels of coverage (*C* =50% and *C* =75%), and *G*_*Foraging*_, with low level of coverage (*C* =25%), are applied in isolation. Both HVCIs, in combination, are applied with low level of coverage (*C* =25%) each. In all cases, killing *K* is fixed at a low level of 25%. Each colour-coded plot represents a specific case of isolated or combined application, with colour keys presented in the legend. The x-axis denotes simulation time (in days), and the y-axis denotes abundance or PIF. The warm-up period is omitted from the results.

The results of applying HVCIs in isolation and in combination are shown in Figures 7 and 8. Figure 7 depicts the impact of applying *BMS*_*Foraging*_ and *BMD*_*Resting*_ (which correspond to ITNs and IRS, respectively) with varying levels of coverage (*C*). Both HVCIs are applied first in isolation with *C* =50% and *C* =75%, and then in combination with *C* =50% each, with killing *K* being fixed at 50%. As shown in Figure 7a, the combined case of *BMS*_*Foraging*_, *BMD*_*Resting*_, *C* =50% reduces abundance by 30 and 15% more than those obtained by the isolated cases of *BMS*_*Foraging*_, *C* =50% and *BMD*_*Resting*_, *C* =50%, respectively. With PIF, as shown in Figure 7b, the combined case achieves approximately 97% reduction. In both populations, an interesting synergistic effect is also observed: the combined case, with *C* =50%, performs better than both HVCIs with higher coverages (*C* =75%).

Figure 8 depicts the impact of applying *BMD*_*Resting*_ and *G*_*Foraging*_ (which correspond to IRS and lethal ovitraps, respectively). *BMD*_*Resting*_, with moderate and high levels of coverage (*C* =50% and *C* =75%), and *G*_*Foraging*_, with low level of coverage (*C* =25%) are applied in isolation, and compared to the combined application of *BMD*_*Resting*_, *G*_*Foraging*_, *C* =25% (i.e., with low coverage). In all cases, killing *K* is fixed at a low level of 25%. As shown in Figure 8a, even with low coverage, the combined case performs better than the isolated cases (each with higher coverage) in reducing abundance. With PIF, as shown in Figure 8b, the combined case achieves approximately 90% reduction, and performs better than all isolated cases.

These results point to some interesting observations. First, targeting multiple stages simultaneously by an HVCI with low coverage can be more effective than targeting the same stages by multiple HVCIs (applied in isolation) with comparatively higher coverages. Second, when multiple HVCIs are combined, some synergistic impacts are observed, as shown in Figures 7 and 8; however, these combined impacts are additive (and not multiplicative), and is more effective with higher coverages, confirming similar findings in [4].

## Discussion

Some of the key features, characteristics and limitations of the core model are highlighted below.

### An extendible framework for other anopheline species

Although the model described in this paper has been designed specifically around the mosquito *An. gambiae*, a wider range of other anopheline species with different behaviours or biology can also be incorporated into the model. For example, in the current model, the host seeking mosquitoes are assumed to be uniformly anthropophilic, and alternative hosts for blood-feeding (e.g., cattle) are not modelled. By modifying the host seeking assumptions in the BMS stage, the zoophilic behaviour of other species can be easily modelled.

Abundances of most anopheline species are profoundly affected by a variety of factors, including weather (e.g., temperature, rainfall, humidity, seasonality cycles) and habitat (e.g., habitat size, surface area, habitat characteristics). In order to adapt other species, some of these factors also need to be included with finer details. The model currently captures the effect of all weather factors by using a single variable (combined seasonality factor *r*, see Methods), and does not explicitly include various habitat factors. However, it provides an excellent framework for such extensions in the future.

### Exploring other research problems

The utility of the core model is also demonstrated by several other extensions (published elsewhere). For example, it is extended to have an explicit spatial representation by appending spatial properties to the mosquito agents and the underlying landscapes [4, 5]. Using the spatial model, the individual and combined impact of two real-world interventions (LSM and ITNs) are investigated, and then are qualitatively compared to the results reported by other studies. Other applications of the core model include evaluating the effectiveness of evolution-proof, late-life-acting, and instant-acting insecticides [48], and exploring the impact of various SIT strategies on a simulated mosquito population [49]. These applications, each of which investigates a specific set of well-defined biological research questions, can be treated as extensions of the core model.

### HVCIs

Among the four HVCIs that target the adult stages, the impact of *IA*_*Resting*_ proves to be the least effective. With varying coverages and varying temperature ranges, the three HVCIs *BMS*_*Foraging*_, *BMD*_*Resting*_, and *G*_*Foraging*_, which target the gonotrophic cycle stages (BMS, BMD and G, respectively), produce higher impacts than the rest, with *G*_*Foraging*_ being the best in reducing the PIF mosquito populations. This can be attributed to the facts that these HVCIs include both multi-hour mortality impacts (usually of much longer durations than the rest), as well as impacts at each repeated gonotrophic cycle. Since they are able to impose additional killing effects for much longer durations, they perform progressively better with higher coverages. Thus, the G stage may be the most effective point of target for the newly developed and novel interventions (e.g., the lethal ovitraps). These observations also indicate the importance of designing the appropriate interventions that can target the gonotrophic cycle stages for an effective control or interruption in the transmission cycle.

In the low-mid coverages and temperature ranges, *L*_*Updating*_ performs better than the rest, especially in reducing the adult female populations. Although being applied only once in the mosquito’s life cycle, its impact increases as coverage increases, as a consequence of being applied for comparatively more number of time steps in the longest-duration larval stage.

In the mid-high temperature range, the impacts of *L*_*Updating*_, *IA*_*Resting*_, and *BMD*_*Resting*_ on both populations (adult females and PIFs) are gradually diminished. In higher temperatures, developments in these temperature-dependent stages happen must faster. As a result, durations of these HVCIs are shortened, and their impacts are reduced. For the same reason, higher temperatures also increase the overall mosquito populations significantly.

The two larval HVCIs (*L*_*Entering*_ and *L*_*Updating*_) are separated in order to capture the intricate details entailing different forms of LSM. *L*_*Updating*_ represents various conventional methods of LSM (e.g., habitat modification, habitat manipulation, biological control, larviciding) that prevent the completion of immature development with the goal of permanent elimination of targeted habitats. On the other hand, *L*_*Entering*_ represents various abstract methods of other possible interventions with non-cumulative impacts, which may not directly translate to existing real-world interventions (it is included in the model for completeness). For example, *L*_*Entering*_ may target a habitat in which eggs are laid and hatched, but some chemical kills a portion of the eggs (as opposed to hindering the larval development). However, this separation (between *L*_*Entering*_ and *L*_*Updating*_) may be unrealistic in most real-world scenarios.

*L*_*Entering*_, even with high coverage and moderate killing, has little impact on the population. Since it imposes a one-time killing effect when an egg enters the L stage, it may not capture the significant impact of LSM that has been observed in many studies.

Although the impact of *L*_*Updating*_ appears to be very high, it is likely to be significantly overestimated in the model for several reasons. First, it does not consider any density-dependent reduction in mortality. Next, it is treated as a continuous (as opposed to time-limited) mechanism that imposes additional mortality during the entire simulations. In the real world, these assumptions may not be valid for most scenarios. For example, most larval habitats can be flushed away due to high rainfall and/or predation [47], reducing the impact of any pre-existing larval interventions. Next, the definition of coverage *C* (as adopted by the core model) implies the probability of encountering an HVCI in a particular stage (unlike other models, the related parameters of distribution and usage are not differentiated for *C*). Thus, for the L stage, *C* denotes the proportion of anopheline habitats that are covered or treated by *L*_*Updating*_, and a larva in an untreated habitat would not be exposed to this factor (unlike adults which may move for host seeking from one house to another, and therefore can be exposed at some point even if *C* is low). Therefore, in reducing the adult populations (by more than 25%), the impacts of *L*_*Updating*_ are likely to be overestimated.

The HVCIs that simultaneously target multiple life cycle stages perform better due to their multi-hour mortality applications applied over much longer durations. Also, when several HVCIs are compared in isolation and in combination, a combined HVCI with low coverage produces additive synergistic impacts and can be more effective than isolated HVCIs with comparatively higher coverages.

In the current model, the HVCIs ignore other related factors such as excitorepellency by bed nets. Therefore, any potential community effect for untreated nets at high coverages (which has been observed by other studies) may not be observed by the current model. However, such effects can easily be explored/reconciled by the spatial extension of the current model, which modelled excitorepellency by ITNs by including a repellence parameter R, and discussed its effects at length [4].

### Miscellaneous issues

The core model implements density- and age-dependent larval mortality and age-dependent adult mortality rates. Density- and age-dependent larval mortality incorporates the combined effects arising from the age of larvae, and other important factors in the larval population that include predation, cannibalism, resource competition. By keeping track of the one-day old equivalent larval population and the age-adjusted biomass in an aquatic habitat, the model provides a check and balance mechanism to regulate the habitat’s capacity and to maintain a soft upper limit for the aquatic population. Age-dependent adult mortality (vector senescence) allows the model to capture the age-dependent aspects of the mosquito biology, and thus provides a more realistic foundation to examine, in the future, other important determinants of the pathogen transmission cycle (e.g., biting rate, host preference, vector competence, dispersal, resistance to insecticides), which have been reported to be affected by vector senescence [37–39].

The selection of an hourly timestep (as opposed to a daily timestep) allows the ABM to capture multiple contingent events occurring during the nights more realistically. For example, it allows a mosquito agent to oviposit and find a blood meal during the same night at different hours, instead of waiting for (at least) two consecutive nights to perform the same.

The ABM provides a very convenient framework to explore the impacts of targeting specific activities and combining multiple HVCIs. As some of the theoretical coverage and efficacy values of the HVCIs are translated to real-world interventions, it may be easier to achieve a desired level of killing or coverage for some intervention modality than another. For example, it may be easier to achieve higher killing and coverage levels for IRS than for ovitraps. Thus, a 50% killing does not necessarily translate in terms of equal feasibility for these real-world interventions.

The combined seasonality factor *r* is used to modify the habitat capacity, as shown in Equation 8 (see Methods). It can be adjusted in different models with different seasonality settings (e.g., to model one rainy season vs. year round transmission). Results presented in this paper do not include such variations.

All simulation results presented in this study represent the averages of at least 30 replicates. However, for certain elimination scenarios, 30 replicates may not necessarily be enough to adequately map out the stochastic effects (although none of the results were affected by this issue). In such cases, rigorous testing should be performed to identify the adequate number of replicates, the importance of which was demonstrated previously using an earlier version of the current ABM [4].

In the simulations, the 100-day warm-up period ensures that the model has reached steady state, and should not be treated as an absolute value. Each generation of the mosquitoes requires ≈ 15 days to become mature, and it takes ≈ 2-3 generations for the initial model to reach equilibrium. Thus, a 50-day warm-up period would have been sufficient in most cases. However, the 100-day warm-up period also guards against oscillatory spikes in the abundance, which may occur due to several factors such as generation-to-generation oscillation tendency, density-dependence and skip-oviposition effects, short hiatus in egg-laying, etc.

## Conclusions

A conceptual entomological model of the population dynamics of *An. gambiae* and the agent-based implementations derived from it are described. Although the model is designed specifically around the mosquito *An. gambiae*, it could effectively apply to a wide range of other anopheline species with different behaviours by incorporating a variety of biological and seasonal factors. Thus, the model can essentially be treated as a generic *Anopheles* model, offering an excellent framework for such extensions. The density- and age-dependent mortality rates (vector senescence) allow the model to capture the age-dependent aspects of the mosquito biology and provide a more realistic foundation to examine other important determinants of the pathogen transmission cycle in the future. The utility of the core model has also been demonstrated by several other applications, each of which investigates a specific set of well-defined biological research questions across a variety of dimensions (including spatial models [4, 5], insecticide resistance [48] and SIT [49]).

The results of all simulations presented in this paper should be interpreted as qualitative and relative, as opposed to quantitative, until future data, obtained by field and laboratory studies, can be used to parameterize, calibrate and validate the ABMs.

It is also emphasized that researchers worldwide can better utilize the value of such modelling efforts by integrating the models within an open-access execution environment. The Vector-Borne Disease Network (VecNet) [50], for example, may provide a suitable framework to store and use such models. A variety of models, including the individual-based model *EMOD* [51] and the *OpenMalaria* epidemiology model [52], are already integrated within VecNet. These models can all benefit by gaining high visibility and practical use from VecNet’s digital library, integrated modelling platform and shared execution environment. This, in turn, may deliver greater impact to a wide range of user communities of these models.

## Abbreviations

ABM: Agent-based model
ATSB: Attractive toxic sugar bait
BMD: Blood meal digesting
BMS: Blood meal seeking
DMR: Daily mortality rate
E: Egg
G: Gravid
GLMM: Generalized linear mixed model
GMM: Genetically modified mosquito
HC: Habitat capacity
HMR: Hourly mortality rate
HVCI: Hypothetical vector control intervention
IA: Immature adult
IRS: Indoor residual spraying
ITN: Insecticide-treated net
IVM: Integrated vector management
L: Larva
LSM: Larval source management
LLIN: Long-lasting insecticidal net
MS: Mate seeking
ODE: Ordinary differential equation
P: Pupa
PIF: Potentially infectious female
RIDL: Release of insects carrying a dominant lethal
SIT: Sterile insect technique
TBV: Transmission-blocking vaccine
VecNet: Vector-Borne Disease Network
WHO: World Health Organization.
